# Chloroplast DNA methylation in the kelp *Saccharina latissima* is determined by origin and possibly influenced by cultivation

**DOI:** 10.1111/eva.13744

**Published:** 2024-07-02

**Authors:** Lydia Scheschonk, Anne M. L. Nilsen, Kai Bischof, Alexander Jueterbock

**Affiliations:** ^1^ University of Bremen, Marine Botany & MARUM Bremen Germany; ^2^ Algal and Microbial Biotechnology Division, Faculty of Biosciences and Aquaculture Nord University Bodø Norway

**Keywords:** aquaculture, epigenetics, marine algae, marine macrophyte, non‐model organism, organelle genome methylation, plastid

## Abstract

DNA cytosine methylation is an important epigenetic mechanism in genomic DNA. In most land plants, it is absent in the chloroplast DNA. We detected methylation in the chloroplast DNA of the kelp *Saccharina latissima*, a non‐model macroalgal species of high ecological and economic importance. Since the functional role of the chloroplast methylome is yet largely unknown, this fundamental research assessed the chloroplast DNA cytosine methylation in wild and laboratory raised kelp from different climatic origins (High‐Arctic at 79° N, and temperate at 54° N), and in laboratory samples from these origins raised at different temperatures (5, 10 and 15°C). Results suggest genome‐wide differences in methylated sites and methylation level between the origins, while rearing temperature had only weak effects on the chloroplast methylome. Our findings point at the importance of matching conditions to origin in restoration and cultivation processes to be valid even on plastid level.

## 1 INTRODUCTION

In the face of the staggering changes in local and global ecosystems due to the anthropogenic climate crisis, gaining knowledge in the field of eco‐evolutionary dynamics is crucial. Due to the unique contribution of chloroplasts to life, it is vital to understand genetic as well as epigenetic processes in these organelles. Processes of photosynthesis within the chloroplasts depend on both the nuclear genome and the chloroplast genome, as the organelles depend on extra‐organellar products for their internal processes due to the reduced nature of their genome (Wang, Leister, & Kleine, 2020; Wang, Wang, et al., 2020). In both, the nuclear and the chloroplast genome, regulation via epigenetic mechanisms has been observed in plants and algae (Finnegan et al., 1998; Wang, Leister, & Kleine, 2020; Zhang et al., 2018). These processes or mechanisms include histone modification, non‐coding RNA and DNA methylation (Boquete et al., 2021). They have been shown to play vital roles in many cellular processes, such as regulation of gene expression, mRNA processing, timing of DNA replication, determination of chromatin structure or silencing of transposons (Finnegan et al., 1998; Zhang et al., 2018). Epigenetic processes hence hold the key to rapid local adaptation, and the capacity for species survival and ecosystem stabilization despite swift alterations in biotic and abiotic factors. DNA cytosine methylation is the most stable among the currently known epigenetic mechanisms, and can persist for multiple generations (Lämke & Bäurle, 2017), thus is presumed to be most relevant for epigenetic adaptation. In contrast, histone modifications and ncRNAs are often only stable for several days or weeks (Lämke & Bäurle, 2017). In primary producers, the chloroplast genome has long been presumed to be generally un‐methylated, despite data suggesting otherwise (*Saccharina japonica*, Teng et al., 2021; *Chlamydomonas reinhardtii*, Nishimura, 2010). The function of chloroplast genome methylation is yet largely unclear, but intensely researched. The few species of mostly agricultural use that show chloroplast methylation, such as tomato and rice, or the unicellular green alga *Chlamydomonas* (Muniandy et al., 2019; Niederhuth et al., 2016; Nishimura et al., 1999; Wang, Leister, & Kleine, 2020), typically have been analysed via methods that fall under the umbrella of whole genome bisulphite sequencing (WGBS; Muniandy et al., 2019; Nishimura et al., 1999). A recent method for non‐model organisms that captures methylation in CG and CHG sequence contexts is the MethylRAD method (Wang et al., 2015), which since has broadened methylome research (Dixon & Matz, 2020a, Dixon & Matz, 2020b: marine invertebrates and reef‐building coral; Jueterbock et al., 2020: Seagrass; Saha et al., 2020: differentiating embryonic stem cells; Scheschonk et al., 2022: kelp; YanJun et al., 2021: potato; Niu et al., 2022: viviparous black rockfish; Xu et al., 2022: leaf spots in wheat). These studies collectively highlight the versatility and effectiveness of the MethylRAD method in various biological research areas. MethylRAD provides single‐base resolution and, although not covering the entire methylome, it has the potential to reveal genome‐wide DNA methylation patterns consistent with WGBS, but at a fraction of the cost and required DNA inputs.

Still, knowledge is scarce regarding the function of plastid methylomes. The main hypotheses include the regulation of maternal inheritance of the organelles (Burton et al., 1979), as well as protection of the nuclear genome via plastid DNA methylations (Yoshida et al., 2019). In marine primary producers, the first chloroplast genome methylation has recently been documented in the brown macroalga *S. japonica* (Teng et al., 2021). As in plants, its functional relevance remains unknown. Even the inheritance of the chloroplast genome is not consistent among species. In nearly all eukaryotes, most individuals inherit plastid genes from only the maternal parent (Birky, 1995). In (oogamous) brown algae, the majority of evidence supports the claim that chloroplasts are primarily maternally inherited (Choi et al., 2020; Motomura et al., 2010). The algal pattern of maternal inheritance is consistent with that observed in most land plants and green algae (Choi et al., 2008). However, while maternal inheritance seems prevalent in general, there may be exceptions to this pattern in certain organisms. Bi‐parental inheritance of chloroplasts, for example, has been reported in some isogamous brown algae (Kato et al., 2006 *Scytosiphon lomentaria*; Liang et al., 2022 Ectocarpales species).

While epigenetic mechanisms have been extensively researched in terrestrial plants, knowledge about macroalgal or kelp epigenetics still is limited to a handful of studies (Cock et al., 2010; Fan, Han, et al., 2020; Fan, Xie, et al., 2020; Scheschonk et al., 2022), with a single study on chloroplast DNA methylation (Teng et al., 2021). However, macroalgae are the predominant source of coastal marine primary production (Field et al., 1998; Filbee‐Dexter, 2020). They are nearly exclusively restricted to the shorelines of the continents, where they act as foundation species of marine rocky shore ecosystems, and form the base of food webs, hence, have high and diverse ecological value (Bartsch et al., 2008; Duffy et al., 2019; Teagle et al., 2017). The economic value of kelp and macroalgae in general is increasing exponentially especially in the western world, as macroalgae cultivation gains momentum outside of Asia as an environmentally sustainable new blue economy with a multitude of applications (Cai et al., 2021). In 2019, marine macroalgae accounted for ~30% of the global production of marine aquaculture commodities (120 million t), worth US$ 14.7 billion (Cai et al., 2021). However, in recent years, macroalgae distributions have shifted northward in response to increasing (summer) water temperatures (Bringloe et al., 2020; Krause‐Jensen et al., 2020). Heat waves affect both wild populations and aquaculture yields. In kelp, the respective thermal tolerance of gameto‐ and sporophyte stages seems to be the strongest factor defining their global distribution limits (Liesner et al., 2020; Monteiro et al., 2019). Temperature is a controlling factor in many biological processes such as photosynthetic carbon acquisition. Enzymatic activity of RubisCO, the carbon capturing enzyme in photosynthesis, increases by a factor of 7 between 5 and 15°C (Cen & Sage, 2005), and has been shown to be especially vulnerable to high temperatures. As the chloroplast genome carries several photosynthesis‐relevant enzymes, investigating its methylation at different temperatures is particularly relevant when considering methylome‐based temperature adaptation.

Our study is the first attempt to determine the influence of temperature, origin, and growth condition (wild vs. cultivated) on the methylome of chloroplasts of the kelp *Saccharina latissima* (sugar kelp). In the northern hemisphere, this boreal‐temperate marine brown alga, a congener species to *S. japonica* with a large latitudinal distribution range, is among the macroalgal species of both high ecological and economic value (Bartsch et al., 2008; Cai et al., 2021; Diehl et al., 2024).

The aims of our study were

- To determine the existence of a methylome in the chloroplast of *S. latissima*, as has been reported in *S. japonica* (Teng et al., 2021).
- To characterize whether maternal inheritance decouples the chloroplast methylome variation from the nuclear genome variation.
- To identify whether fertilization and growth under contrasting temperatures can create methylome shifts that resemble methylome differences between origins with contrasting thermal histories.

## 2 MATERIALS AND METHODS

### 2.1 Laboratory cultures

Clonal gametophyte cultures of *S. latissima* (Linnaeus) C.E. Lane, C. Mayes, Druehl & G.W. Saunders from Helgoland, German Bight (Nordstrand, 54°11′18.9″ N 7°54′14.1″ E; HG1, HG2, HG3, HG4 in culture since 2014) and Spitsbergen, Svalbard (Hansneset, Kongsfjorden, 78°59′26.0″ N 11°58′42.3″ E; SG1, SG2, SG3, SG4 in culture since 2015) were mono‐parentally fertilized. Gametophytes were kept at resting stage under identical conditions. As all gametophytes within a respective culture (e.g. HG1) were grown as clones stemming from the same zoospore (which is a single cell), all gametophytes within this culture (e.g. HG1) can be presumed to be true clones. In the resulting sporophyte cultures (Helgoland: HG1♀ X HG1♂ = H1, HG2♀ X HG2♂ = H2, HG3♀ X HG3♂ = H3, HG4♀ X HG4♂ = H4; Spitsbergen: SG1♀ X SG1♂ = S1, S2♀ X S2♂ = S2, S3♀ X S3♂ = S3, S34 X S4♂ = S4; see Figure S5), all sporophytes within a culture (e.g. H1) were twins (but not clones anymore), as meiotic processes (such as chromosome recombination) occur.

Sporophyte cultures were initially kept for ≥6 weeks in petri dishes in autoclaved natural seawater (Salinity 34) that was Provasoli‐enriched (PES) with half the concentration used for grown sporophytes (=½ PES). As early as fertilization was initiated, the four cultures per origin (S1–S4, H1–H4; technical replicates) were kept at each of the three temperatures (5, 10 and 15°C) under white light at 15 μmol m^−2^ s^−1^ (18:6 h light:dark). PES in petri dishes was changed every ~10 days. During each water change, only the largest individuals were maintained in culture, while smaller plants were discarded. Growth rates between the temperatures, but not origins, differed strongly. When the sporophytes in a petri dish reached a length of about 2 cm, they were transferred to aerated 1 L –Schott bottles (with sterile ½ PES; five plants per bottle). At a length of about 5–7 cm, ½ PES was changed to full PES, exchanged at least every 7 days. At a length of 12 cm, sporophytes were moved to aerated 3 L—beakers with sterile full PES, which was exchanged at least once a week.

From each culture, sporophytes of corresponding size (>4 per beaker) were frozen at −80°C. After all samples had been obtained, they were placed into cellulose bags, the bags covered in silica gel to dry; only sporophytes with a fresh weight exceeding 120 mg (without stipe) were taken for DNA extraction. One dried sporophyte per beaker was randomly chosen as sample for extraction. In three beakers, no sporophytes had developed successfully, so in total 21 instead of 24 laboratory samples could be sequenced.

### 2.2 Field samples

Adult sporophytes were sampled from Helgoland, German Bight (54°11′18.9″ N 7°54′14.1″ E) during the last week of May 2019 (*n* = 10, ~10°C at 10 m depth at time of sampling; annual ~max/~min: ~19°C/~5°C; see Figure S6), and from Spitsbergen, Svalbard (78°59′26.0″ N 11°58′42.3″ E) during the last week of June 2019 (*n* = 10, ~5°C at 10 m depth at time of sampling; annual ~max/~min: ~7.5°C/~−1.4°C; see Figure S7), at the same locations from which the gametophytes for the laboratory grown sporophytes originated. As seasons, and with this, circannual processes, are shifted in timing in the high Arctic relative to temperate regions, we presume the difference in sampling time to be negligible.

The same scuba diver sampled on Helgoland and Spitsbergen. At each site, 10 sporophytes were retrieved from corresponding depths. From each sporophyte, 4 discs (ø = 3 cm) were cut from the phylloid, omitting the meristematic region (very young, non‐representative tissue), and ‘midsection’ (location of differentiated cells for assimilate transport along sporophyte, and (when present) reproductive tissues; for morphological display, see Diehl et al., 2024). All tissue samples (laboratory and field) were dried in cellulose bags in silica. Prior to DNA extraction, all pre‐dried samples (laboratory and field) were placed in an oven and dried overnight at ~45°C. In total, 21 samples from laboratory culture, and 20 samples from the wild were analysed. In contrast to the field samples, our laboratory samples were uniparentally fertilized. However, chloroplast genes are transmitted solely from the maternal parent (matrilineal; Kuroiwa, 1991, in Nishimura, 2010). Thus, the chloroplast genome inheritance can be presumed identical in uniparentally and bi‐parentally fertilized individuals. This circumstance enables a direct comparison between the chloroplast methylomes of the cultured and wild samples.

### 2.3 Bioinformatics and statistics of MethylRAD sequencing

#### 2.3.1 Clean‐up of raw data

DNA was extracted and MethylRAD sequencing libraries (following Wang et al., 2015) were prepared as described in detail in Scheschonk et al. (2022). The sequences were de‐multiplexed by sample and quality trimmed with TrimGalore! v 0.4.1 (https://www.bioinformatics.babraham.ac.uk/projects/trim_galore/). Bases with Phred‐score < 20 (‐‐quality 20) were eliminated, the adapter sequence was removed (‐‐stringency 3), and the terminal 2 bp (both ends) were removed to eliminate any artefacts at the ligation position (‐‐clip_R1 2\‐‐three_prime_clip_R1 2). Reads were checked for fragment (over‐) representation, base AT CG content bias, and read length with FastQC v0.11.8 before and after quality trimming.

#### 2.3.2 In silico digestion

For an overview of potential methylation sites, and as a backbone to map sequences to the reference chloroplast genome for the *S. latissima* chloroplast (Fan, Xie, et al., 2020) was digested in silico using the custom python script InSilicoTypeIIbDigestion_corrected.py (http://marinetics.org/2017/04/11/REdigestions.html) and settings for simulating the restriction enzyme FspEI. The in silico digestion found 270 recognition sites, where 69 were CCGG recognition sites, 61 CCTGG sites, 77 CCTGT sites and 63 CCAGG sites.

#### 2.3.3 Mapping and annotation

The sequenced reads were mapped to the in silico digested reference genome of the *S. latissima* chloroplast, combined with the in silico digested genome of the closely related species *S. japonica* to account for reads belonging to the nuclear genome and avoid false mappings to the chloroplast genome for those reads. Reads were mapped using Burrows‐Wheeler Aligner (BWA Version: 0.7.17‐r1188) (Li & Durbin, 2010). Duplicate mappings were excluded from further analyses by filtering out reads with a mapping quality <10 using samtools (v 1.9). A count table was created listing the number of reads that mapped back to each methylation site. For this, htseq‐count (v 0.7.2) was called on each sample alignment with in silico digested fragment in gff3 format as count features. An additional table was made with counts normalized to ‘reads per million (RPM) = (Reads mapped per site × 10^6^)/(Total number of mapped reads)’ to account for varying sequencing depths.

Only methylated sites that were covered in at least one library with coverage >2 were kept in count tables and RPM tables. The sites were annotated for their sequence context (CG or CHG), genomic region, gene ID numbers and gene functions based on the gff3 annotation file for the *S. latissima* chloroplast genome from NCBI (Accession: MT151382.1) (Fan, Xie, et al., 2020).

### 2.4 Statistics

#### 2.4.1 Principal component analysis

Differences in the chloroplast methylome were investigated with principal component analyses (PCA) on RPM tables using the R package ‘FactomineR’ (Le et al., 2008). Since laboratory and field samples separated clearly into distinct groups in the PCA, their methylomes were compared first for all samples, and then separated into origins (Helgoland and Spitsbergen). Within the laboratory and field samples, and for all samples, we compared origins (Helgoland and Spitsbergen). Within the laboratory samples, we compared between the treatment temperatures of 5, 10 and 15°C.

#### 2.4.2 Outlier detection

The outlier function in the R package ‘FactoInvestigate’ (Thuleau & Husson, 2020) detected two outliers, FH1 and FH9 (Helgoland field samples) in the PCA. The outliers showed few reads mapping to the genome, few methylated sites and high RPM values for some of the methylated sites (Figure S1). Thus, FH1 and FH9 samples were excluded from all follow‐up analyses. No outliers were found in the Spitsbergen samples. As both outliers belonged to the same group (Helgoland field samples), outlier removal led to a reduction in variance explained by the first two principal components from 69.9% to 28.9% (Figures S1–S3).

#### 2.4.3 Methylation levels

We tested for differences in DNA methylation levels as RPM and for differences in the numbers of methylated sites between populations, between treatment temperatures, and between laboratory and field samples, using Wilcoxon rank sum tests. *p*‐values were corrected with the Benjamini–Hochberg method (Benjamini & Hochberg, 1995) to control for the false discovery rate, and results with adjusted *p* values *p*adj < 0.05 were considered significant. The tests were performed using the R package ‘rstatix’ v0.7.0 (Kassambara, 2021).

We tested for correlation between the numbers of quality‐trimmed reads, and the numbers of quality‐trimmed reads that mapped back to the *S. latissima* chloroplast genome (Figures S2c,d and S3c,d).

#### 2.4.4 Differential methylation analysis

We tested for significant differential methylation with the R package ‘DESeq2’ (Love et al., 2014) between ‘Helgoland’ versus ‘Spitsbergen’ in all samples (‘All’ or ‘both locations’), and in only field samples and only laboratory samples. Further, between ‘field’ versus ‘lab’ in all samples, and in only Helgoland samples, and only Spitsbergen samples. We also compared pairwise between temperature treatments: both origins combined (Helgoland + Spitsbergen 5°C vs. 10°C; 5°C vs. 15°C; 10°C vs. 15°C), separately for Helgoland and Spitsbergen (5°C vs. 10°C; 5°C vs. 15°C; 10°C vs. 15°C), and for each temperature (Helgoland vs. Spitsbergen @ 5°C; 10°C; 15°C). ‘DESeq2’ normalized raw methylation counts before testing for differential methylation, and corrected *p*‐values with the Benjamini–Hochberg method for controlling false discovery rates (Benjamini & Hochberg, 1995). Methylated sites with adjusted *p*‐value *p*adj < 0.05 were considered significantly differentially methylated.

#### 2.4.5 GO term analysis

Gene ontology (GO) terms for biological processes, cellular components, and molecular functions were extracted from the database https://www.uniprot.org/ using Gene ID numbers (Tables S2–S4). The R package ‘topGO’ (Alexa & Rahnenführer, 2009) was used to test for enrichment of the GO terms.

#### 2.4.6 Gene names and functions

Names and functions of differentially methylated genes were extracted from the chloroplast genome of *S. latissima* published on NCBI (strain y‐c14; https://www.ncbi.nlm.nih.gov/nuccore/MT151382.1?report=graph).

#### 2.4.7 Analysis of epigenetic distance

Epigenetic distances among laboratory and field samples (cultivation) or among samples from Helgoland versus Spitsbeergen (origin) were estimated as euclidean distances among samples in the PCA. The Shapiro–Wilk indicated non‐normal distribution for groups analysed for cultivation (*p*‐value lab *p* < 0.0001, field *p* < 0.0001) and for origin (Helgoland *p* < 0.0001, Spitsbergen *p* < 0.0001). Hence, we tested for differences in epigenetic distances among the cultivation or origin categories using non‐parametric Wilcox‐rank‐sum test (Table S5).

A Mantel statistic based on Pearson's product moment correlation was conducted in R to assess whether the chloroplast methylome distances are correlated with nuclear methylome distances among all samples.

## 3 RESULTS

### 3.1 The chloroplast methylome

In‐silico digestion identified 69 MethylRAD sites for CG methylation, and 201 for CHG methylation in the chloroplast genome of *S. latissima*. 20.7% and 24.8% of these sites were methylated respectively (Table 1).

**TABLE 1:** Potentially methylated sites for each sequence context, the mean number of methylated sites per sample and standard deviations for the means.

Sequence context	Potential sites (in‐silico)	Methylated sites (mean ± SD)	Methylation level (% mean ± % SD)
CG	69	14.3 ± 1.5	20.7 ± 2.2
CHG	201	49.8 ± 3.0	24.8 ± 1.5

*Note*: Methylation level is the mean percentage of potential methylation sites being methylated in the samples.

Abbreviation: SD, standard deviation.

**FIGURE 1:**
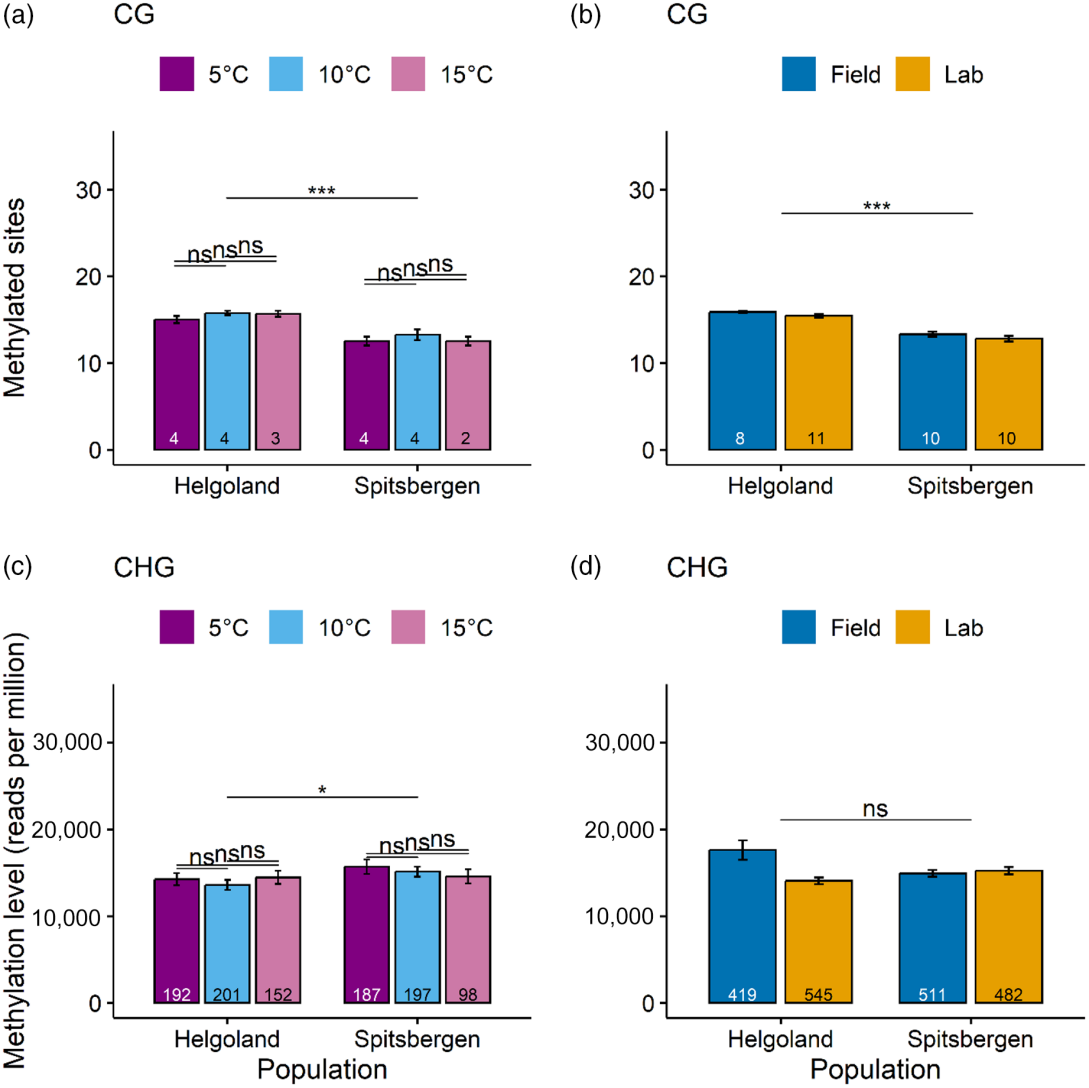
Differences in methylated sites and methylation level between conditions. Methylated CG sites (a, b), and methylation levels as reads per million (RPM) for CHG sites (c, d) between populations and temperatures in laboratory samples (a, c) and compared between origins (b, d). ns, Not significant. Numbers at the bottom of the bars indicate sample size. Significance codes after *p*‐value correction (Benjamini–Hochberg) for multiple comparisons: ‘<0.05’: *, ‘<0.01’: **, ‘<0.001’: ***, ‘<0.0001’: ****.

The number of methylated CG sites was significantly higher in the Helgoland samples than in the Spitsbergen samples both in laboratory samples (*p* < 0.001, Figure 1a) and in laboratory and field samples combined per origin (*p* < 0.0001, Figure 1b). No significant differences were found between treatment temperatures for sites in CHG contexts (Figure S3c). Methylation levels normalized to reads per million (RPM) showed no significant differences in CHG contexts between any groups, but the Spitsbergen population showed higher methylation levels than the Helgoland population in the laboratory samples (*p* < 0.05, Figure 1c).

### 3.2 Principal components of the methylome

**FIGURE 2:**
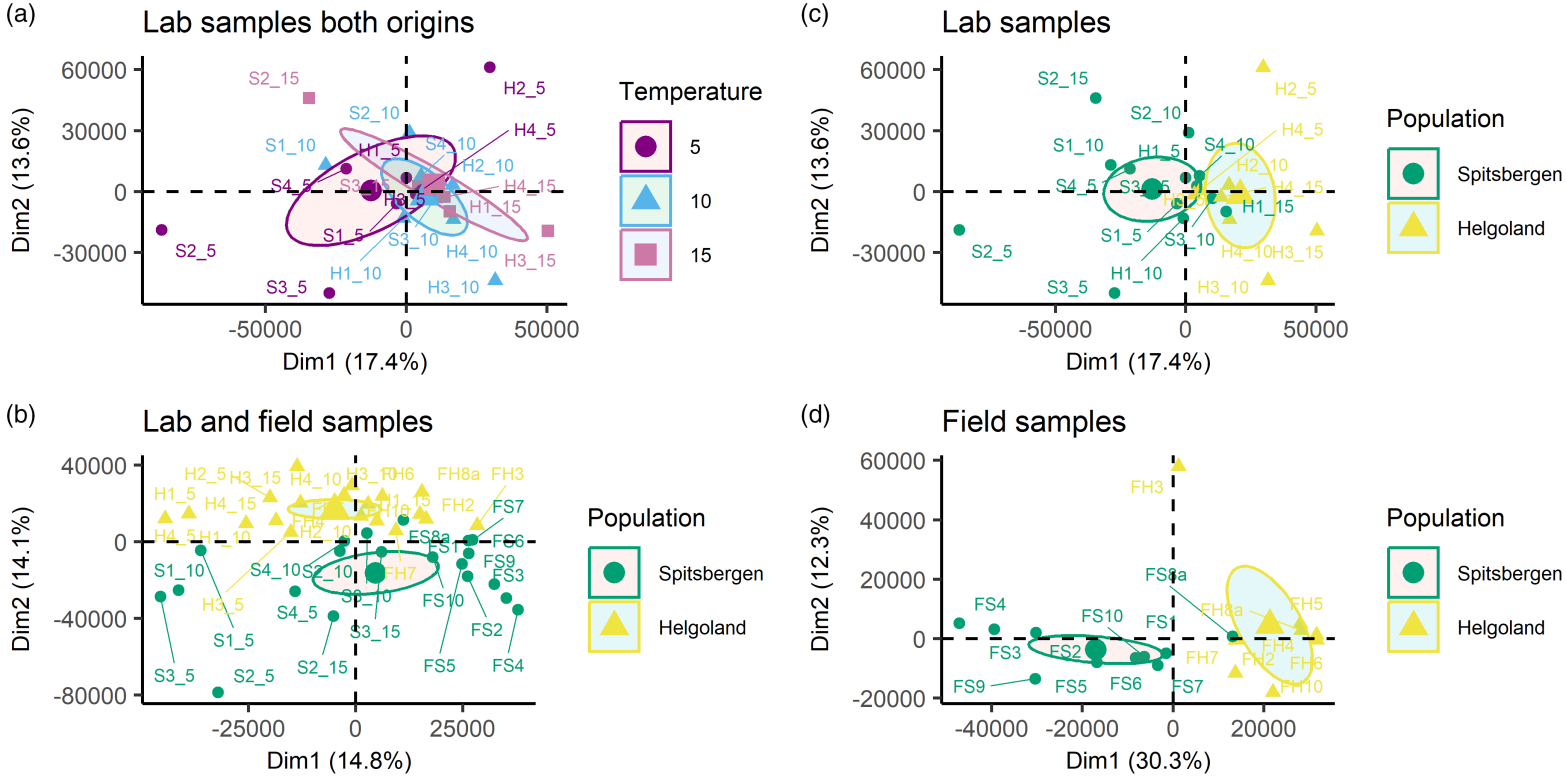
Principal component analysis for (a) Laboratory samples of both origins grouped by temperature treatment. (b) Laboratory samples grouped by origin. (c) All samples, grouped by origin. (d) Field samples grouped by origin. Ellipses represent 95% confidence intervals around the group medians.

Temperatures in the laboratory samples (5, 10, 15°C) did not separate into distinct groups (Figure 2a). The first two principal components equally contributed about 14% to the total sample variation, which was similar between ‘Helgoland versus Spitsbergen’ (Figure 2b). Regarding the origins, the populations of Helgoland and Spitsbergen formed distinct groups along the second principal component when laboratory and field samples were analysed combined (Figure 2b), while when field and laboratory samples were separately analysed for origin, Helgoland and Spitsbergen samples already separated along the first component (Figure 2c,d), with a much stronger influence of origin in the field samples than the laboratory samples (30.3% vs. 17.4%).

### 3.3 Differentially methylated sites and GO terms

**FIGURE 3:**
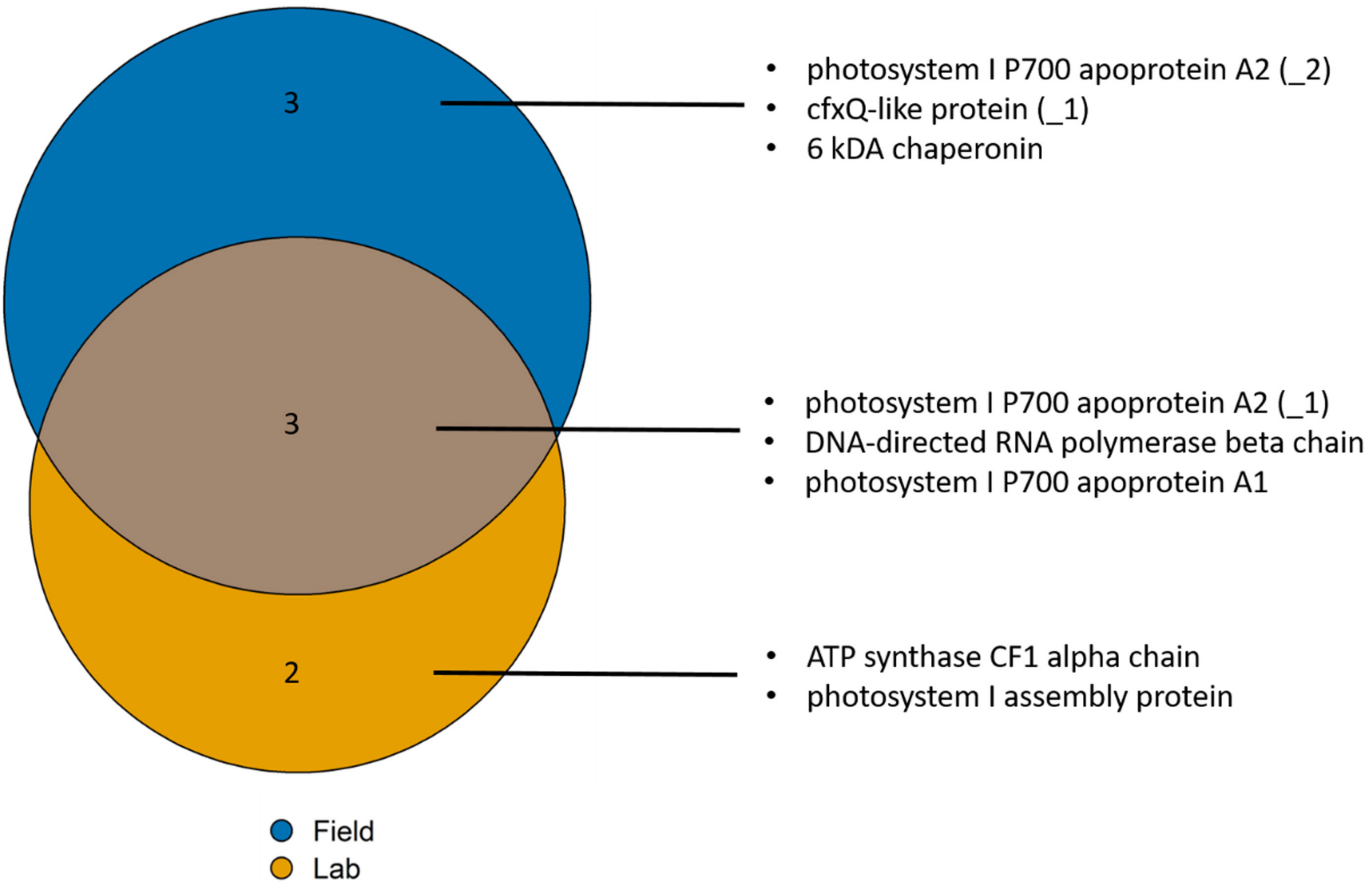
VennEuler diagram showing the numbers of differentially methylated sites between Helgoland and Spitsbergen when analysing field samples (blue), and laboratory samples (orange; Tables S2 and S3). The labels give the product of the genes that were differentially methylated in the respective partitions. (_1) and (_2) depict a gene that showed differential methylation at two different sites. For the exact site position in the respective gene, see Table S6 (‘ST6 Origins’).

Comparing the origins (Helgoland vs. Spitsbergen, Figure 3), differentially methylated sites were as often higher methylated in Spitsbergen samples as in Helgoland samples (Tables S1–S3). Of the eight genes with significant methylation differences between the origins (Figure 3), only three genes (psaB_(1), atpA, yfc3) were identical to the genes with significant methylation differences between the origins at a respective temperature within laboratory samples (Table 2, see colour coding Tables S6 and S7, sheets ‘ST6 Origin’ and ‘ST7 Temperature’).

**TABLE 2:** All conditions that were found to be differentially methylated by the DESeq2 analysis when analysing the different rearing temperatures of the laboratory samples.

Comparison	Gene product	Gene ID
5° vs. 10° Helgoland	**Acetolactate synthase large subunit**	**QKE47546.1**
5° vs. 15° Helgoland	**Acetolactate synthase large subunit**	**QKE47546.1**
5° vs. 10° Spitsbergen	**Photochlorophyllide reductase subunit B**	**QKE47431.1**
5° Helgoland vs. Spitsbergen	Photosystem I P700 apoprotein A2_(1)	QKE47459.1
	ATP synthase CF1 alpha chain	QKE47418.1
	Acetolactate synthase large subunit	QKE47546.1
	Photochlorophyllide reductase subunit B	QKE47431.1
10° Helgoland vs. Spitsbergen	Photosystem I P700 apoprotein A2_(1)	QKE47459.1
	ATP synthase CF1 alpha chain	QKE47546.1
	Photosystem I assembly protein	QKE47473.1
	Photochlorophyllide reductase subunit B	QKE47431.1
15° Helgoland vs. Spitsbergen	Photosystem I P700 apoprotein A2_(1)	QKE47459.1
	ATP synthase CF1 alpha chain	QKE47546.1

*Note*: Most significances were detected between the origins at respective temperature (Helgoland vs. Spitsbergen @ T5/10/15), but only three of those were identical to those detected when all laboratory samples were being analysed as a group (Figure 3, Table S7 (‘ST7 Temperature’)). However, genes QKE47546.1 and QKE47431.1 (bold letters) showed significantly different methylation when analysing for temperature difference as factor (origin, T vs. T).

Two genes were differentially methylated between temperature treatments (Table 2), with ‘acetolactate synthase large subunit’ (QKE47546.1) at two comparisons: 5–10°C (*p*adj < 0.0001), and 5 to 15°C (*p*adj < 0.0017). All other significant differentially methylated genes were found between origins at different temperatures (Figure 3, see colour codes in Tables S6 and S7 (sheets ‘ST6 Origins’ + ‘ST7 Temperature’)).

None of the extracted GOterms was significantly enriched according to the ‘topGO’ enrichment analysis.

### 3.4 Epigenetic distances

**FIGURE 4:**
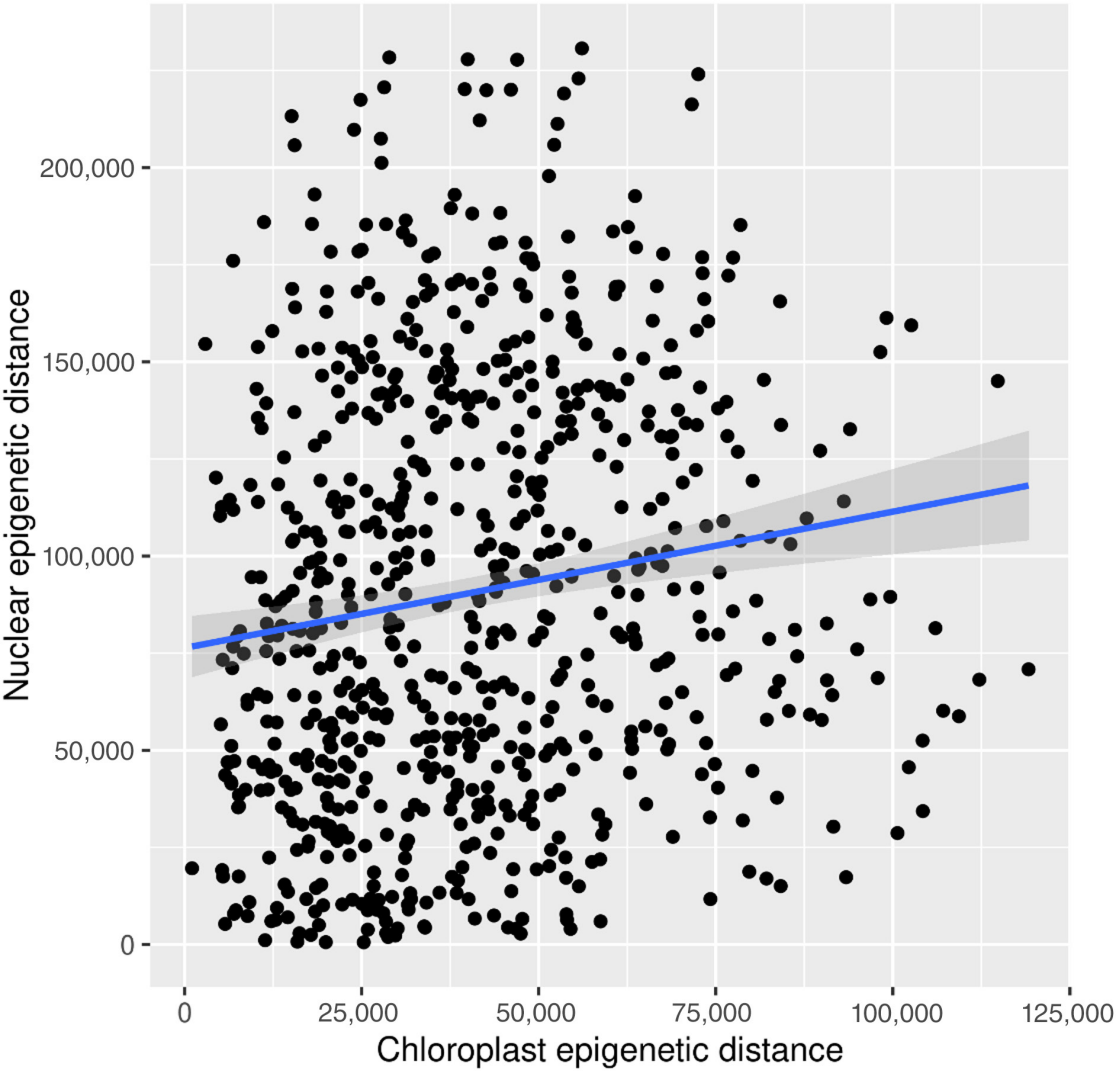
Epigenetic distances in the chloroplast and nuclear methylome (‘genome’). The corresponding mantel test showed that the chloroplast methylome did not in general mirror the nuclear methylome (*r* = 0.145).

**FIGURE 5:**
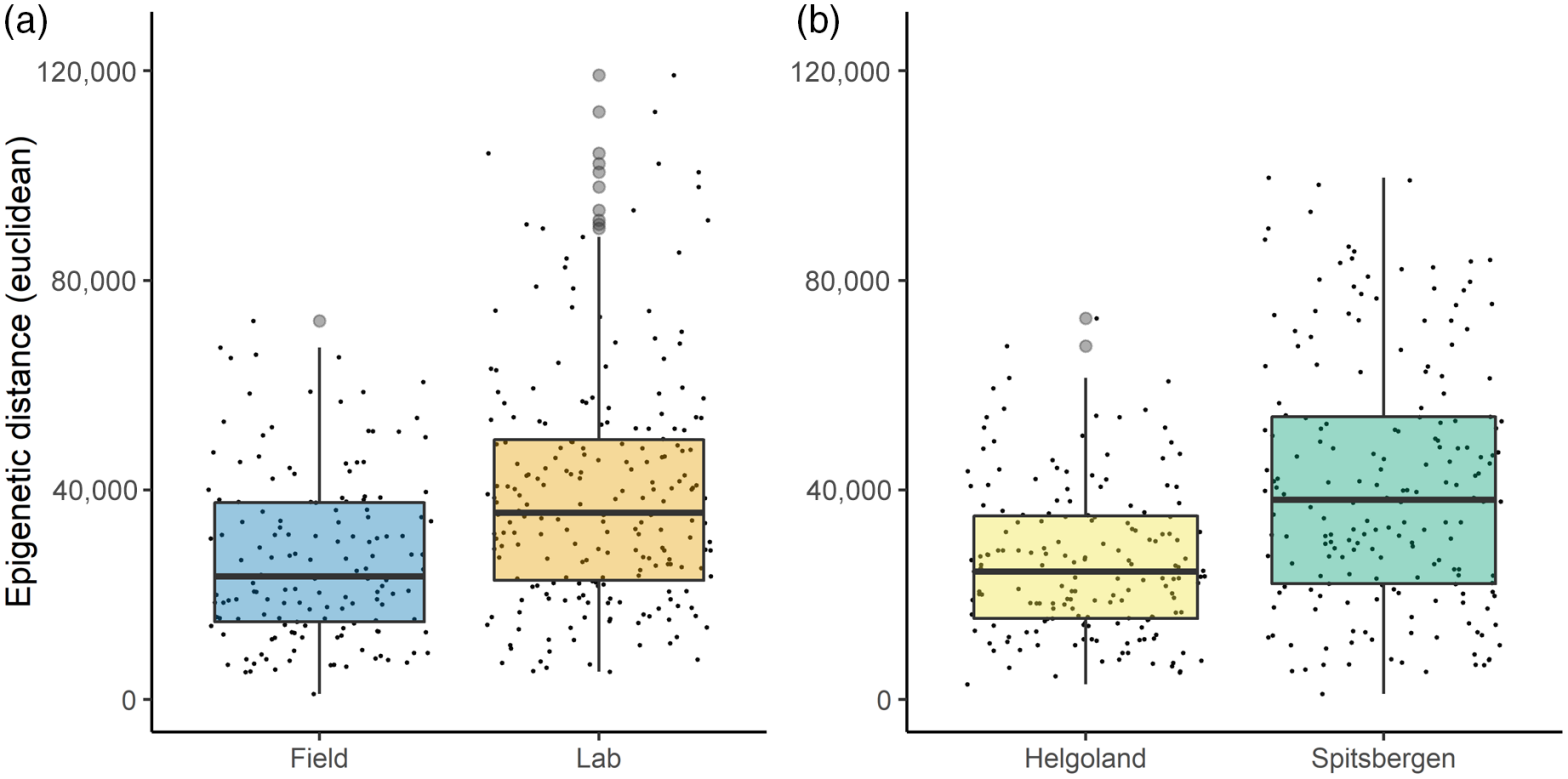
Epigenetic distance (Euclidean distance) for ‘cultivation’, Laboratory (orange) and Field (blue) samples (a), and for ‘origin’, Helgoland (yellow) and Spitsbergen (green) samples (b). The small black dots depict the pairwise distances between the individual samples of each. The light grey dots are outliers detected by the boxplot script but were included during epi‐distance analyses. The boxes represent the interquartile range (IQR) of the respective group, and the horizontal line within the boxes gives the median Euclidean distance.

The Pearson's product–moment correlation between the nuclear and the chloroplast methylomes was significant but low at *r* = 0.145 (Figure 4). In all comparisons (‘cultivation’, ‘origin’, ‘genome’), the epigenetic distances were significantly different between groups (Figure 5). For ‘cultivation’, distances were significantly higher in laboratory samples than in field samples (*p* < 0.0001, median field 153, lab 210), while for ‘origin’ the samples obtained from Spitsbergen showed significantly higher epi‐distances than Helgoland (*p* < 0.0001, median Helgoland 171, Spitsbergen 190; Table S5).

## 4 DISCUSSION

Our study confirms the existence of a methylome in the chloroplast of *S. latissima*, as has been reported in the congener *S. japonica* (Teng et al., 2021; Table 1). We found that maternal inheritance does not limit the establishment of methylome variation, as the chloroplast methylome variation reflected the nuclear methylome variation (Figure 4). For example, both the chloroplast and the nuclear methylomes showed higher methylation levels in Spitsbergen, but more methylated sites in Helgoland (Figure 1; Scheschonk et al., 2022). The strongest methylation differences occurred between origins (Figures 2, 3 and 5, Table 2), which might depend on underlying genetic differences, that we could not capture. At least during fertilization and early sporophyte development, the factor ‘temperature’ appears to be unable to induce differences in chloroplast methylation between origins with contrasting thermal history (Figures 2 and 5, Table 2). We could not show methylome‐wide plasticity in response to temperature variation during early sporophyte development, but rather characterizations of an epigenetic memory. This suggests that the methylome reflects rather long‐term differentiation that may be driven by underlying genetic differences.

### 4.1 Sample origin

The current trend in northward shifts due to heat wave pressure from the south is likely to be counteracted by the long polar nights towards the Arctic Circle (Scheschonk et al., 2019), imposing additional selection pressure. In *S. latissima*, phenotypic variation among populations is often driven by phenotypic plasticity, and the species shows high plasticity in response to changing abiotic factors (Bolton & Lüning, 1982; Diehl & Bischof, 2021).

Population origin had a strong impact on the methylome according to PCA clusters (Figure 2b–d), which was reflected in methylation levels, as well as in the number of methylated sites that significantly differed between origins (Figure 1a,c). Photosystem I (PSI) was the component most affected by differences between the origins, as genes coding for varying aspects crucial to PSI significantly differed at a total of four sites in three genes (Figure 3, Table 2), with the strongest effect on PSI P700 chlorophyll a apoprotein A2. The same gene was one of the two genes that were differentially methylated between the origins in all analysed temperature conditions of the laboratory samples (Table 2, Table S7 sheet ‘ST7 Temperature’). Therefore, the difference may be accounted for by temperature differences among the sample origins. Part of the methylome was origin‐specific even after some years of resting‐stage gametophyte lab‐cultivation. Methylation levels were significantly different between the origins (Figure 1). This may indicate a transgenerational epigenetic memory (Figure 2), although it may be driven by underlying genetic differences. PCA in addition indicated the influence of cultivation on transgenerational methylome differences, as origin played a more pronounced role when analysing laboratory and field samples separately (Figure 2c,d) versus combined (Figure 2b). Even though these are primarily the genes encoded in the chloroplast, differentially methylated genes were exclusively related to functions of photosynthesis (Figure 3), which likely can be explained by the latitudinal differences in the light regimes between origins. Light conditions in the High‐Arctic are strongly seasonal. Photosynthetically active radiation (PAR) is absent for at least 3 months during the arctic winter (polar night), while abundantly available during the arctic summer (polar day). In boreal‐temperate regions, PAR is still seasonal, but available throughout the year. Whether or not the differences observed here are genetically induced cannot be assessed by our set‐up, but the prevalence of the significant difference, detectable in the epigenetic signature that prevailed between origins through cultivation, is a strong indicator for the eco‐genotype approach (Scheschonk et al., 2022). This presumes that adaptation to a specific location is not solely a matter of genetic nature (fixed only in the DNA), but that epigenetic mechanisms play an important role in local adaptation and eco‐evolutionary dynamics of a species (Jablonka & Lamb, 2015; Richards & Pigliucci, 2020). Since methylation and gene expression were shown to be negatively correlated in the *S. japonica* nuclear and chloroplast genes (Fan, Han, et al., 2020; Teng et al., 2021) the origin‐specific methylation of the gene coding for apoprotein A2 (Figure 3, Table S6 sheet ‘ST6 Origin’) may be involved in origin‐specific photosynthetic capacity. A2 is one of the crucial components of PSI. Like with the nuclear genome (Scheschonk et al., 2022) this has implications for cultivation processes as well as restoration efforts, as it shows the importance of suitable origin when considering local growth condition.

### 4.2 Temperature and the methylome

The temperature treatments did not result in distinct clusters in the PCA. Unlike the nuclear methylome, the chloroplast methylome did not differ in methylation levels and numbers of methylated sites in response to the rearing temperatures of 5, 10 and 15°C. However, the different temperature treatments may explain the stronger epigenetic distance among laboratory than among field samples (Figure 5), suggesting that temperature differences to some degree play a role in epigenetic variation, but not as strongly as population origin. This might also indicate a more conserved methylation approach within the chloroplast than the nuclear methylome. Significant differential methylation solely caused by temperature was detected in 2.9% of the genes (Table 2). However, when analysing the origins at equal temperature (Table 2), most differences between origins found at discrete temperatures were in other genes than those detected for the origin analysis. This again might either suggest that the origin‐specific methylome differences are driven by underlying genetic differences, or that an epigenetic memory is at work as the methylomes reacted origin‐specific at discrete temperatures.

### 4.3 Chloroplast and nuclear methylomes

The methylation levels of sites in the chloroplast genome were ca. 100 times higher than levels in the nuclear genome as documented by Scheschonk et al. (2022). In the nuclear genome, the mean methylation level was 0.21% ± 0.13% SD (Scheschonk et al., 2022), while in the chloroplast, the levels were 20.7% ± 2.2% for CH contexts and 24.8% ± 1.5% for CHG contexts (Table 1). The higher methylation level in the chloroplast can be explained by the low numbers of potential methylation sites in the chloroplast genome (Table 1). The pattern of higher methylation in the chloroplast than the nuclear genome is repeated in both *S. japonica* and *S. latissima*, with some dissimilarities that might be caused by different sequencing methods and the inclusion of gametophytes in the studies of *S. japonica* (Fan, Han, et al., 2020; Teng et al., 2021). However, due to the low correlation at high significance (*p* < 0.0009) shown in the mantel test, it can be presumed that the chloroplast methylome does not just mirror the nuclear one (Figure 4).

Photosynthesis is highly sensitive to temperature (Cen & Sage, 2005). However, the main temperature response, in terms of methylation, is observable in the nuclear, but not in the chloroplast genome. The lack of statistically significant differences in the chloroplast genome as compared with the nuclear genome might partly be explained by the lower number of genes. The chloroplast genome has a size of 130 kb and contains 139 protein‐coding genes, three rRNA genes and 29 tRNA genes (Fan, Xie, et al., 2020), while the nuclear genome has a size of 545 Mb and contains 18,733 protein‐coding genes (Ye et al., 2015). However, no significant differences were observed in neither methylation levels nor methylated sites between temperature treatments, despite the ~100 × higher methylation level of the chloroplast. In contrast, in the nuclear genome, a response in methylation levels and numbers of methylated sites to warmer temperature treatments was observed, especially in the Helgoland samples (Scheschonk et al., 2022). The absence of a strong chloroplast methylome shift in response to temperature may be partly explained by the functional importance of the genes, independent from origin or environmental impact, which can be expected to be reflected in a conservative methylome. Another possible explanation for the overall low amount of (differentially) methylated sites among temperature treatments might be that the temperature exposure of the laboratory samples was not stressful for the physiological processes regulated by the chloroplast genome. The difference between the nuclear and chloroplast methylome suggests that between 5 and 15°C the modulation of temperature responses is solely regulated in the nuclear methylome. In *S. latissima*, it has been shown that temperature treatments affect the nuclear methylome, and that differences in the methylome exist between populations from different latitudes, suggesting that epigenetic modifications contribute to local adaptation (Scheschonk et al., 2022). Earlier research on *S. latissima* suggests that short‐term acclimation to different growth temperatures can lead to changes in heat tolerance, and affect photosynthetic performance (Davison et al., 1991), which supports the temperature priming hypothesis suggested for the nuclear methylome (Scheschonk et al., 2022).

In the nuclear genome, most of the differentially methylated sites between laboratory and field samples and between populations were in non‐genic repetitive contexts (transposable elements). In contrast, in the chloroplast genome, the differentially methylated sites were in coding regions of genes, except for very few sites in a promoter region (Tables S2–S4). This is not surprising, given that chloroplast genomes are more condensed and have far fewer noncoding regions than nuclear DNA (Clegg et al., 1994) genomes. Furthermore, it is likely that nuclear methylomes are less conservative, resulting in their higher plasticity.

## 5 CONCLUSION

The chloroplast genome mainly encodes genes with functions involved in processes of photosynthesis, while it is highly depended on the nuclear genome. Processes impacting the chloroplast methylome would have implications for both cultivated and wild populations. Our results showed that sample origin can strongly influence epigenetic mechanisms in the chloroplast, while growth temperature had a minor effect. In cultivars, the importance of matching hatchery and cultivation conditions to origin has been implied by our results, even though there seems to be a tolerance regarding (moderate) temperature. So far, research on differences between origins in *S. latissima* has focused on genetic differentiation, which showed significant genetic variation between populations (Guzinski et al., 2020; Møller Nielsen et al., 2016; Paulino et al., 2016). Epigenetic differences, however, have largely remained unexplored as a source of molecular variation between *S. latissima* populations. As they are likely to play a major role in the chloroplast genome, future studies will need to assess genetic differences controlling the epigenetic responses reported here in detail.

## FUNDING INFORMATION

LS acknowledges funding by Deutscher Akademischer Auslandsdienst (DAAD; research travel grant ‘BremenIdeaOut’ 2019). AN acknowledges funding from Nord University (Master Thesis and transitional stipend). AJ acknowledges funding from Nord University (research talents grant). Parts of this work have been submitted in partial fulfilment of the requirements for Master of Science, at Nord University, Bodo (AN), and Dr. rer. nat, at University of Bremen (LS).

## CONFLICT OF INTEREST STATEMENT

No conflicts of interest were met.

## Supporting information


Data S1:

## Data Availability

Raw sequencing data of this study are available at NCBI SRA BioProject PRJNA809008.
