# Supplementary material for: Chloroplast DNA methylation in the kelp Saccharina latissima is determined by origin and possibly influenced by cultivation
Source: Evol Appl. 2024 Jul 2;17(7):e13744. doi: 10.1111/eva.13744 (PMC11219511; doi:10.1111/eva.13744)
Supplement: Supplementary file 1 — Data S1: [file EVA-17-e13744-s001.zip › supplfile.docx]

# Supplementary

### Suppl. Figures


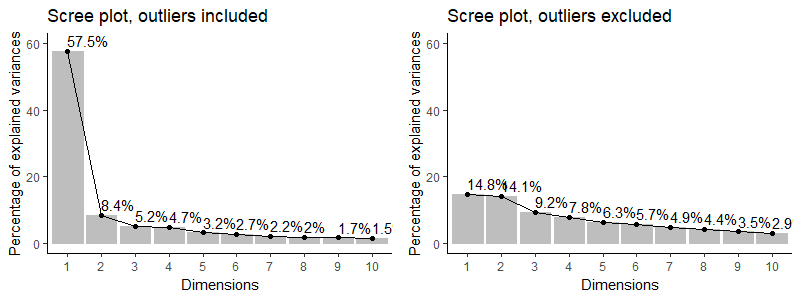


Suppl. Fig. *1*: **Scree plots showing the amount of variance explained by each dimension**. On the left including all samples, on the right when removing two outliers.


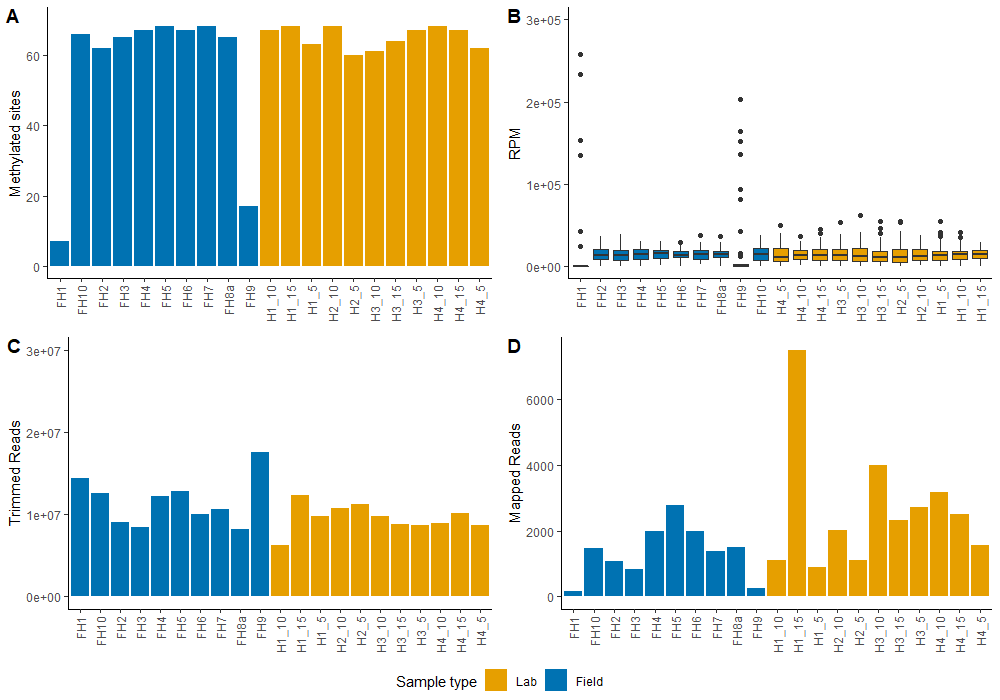


Suppl.Fig. *2*: **Sequencing output and methylation characteristics for each sample from Helgoland.** Plots were used for outlier detection for PCA. A: Number of methylated sites (with more than two reads) for each sample. B: Boxplot showing reads per million (RPM) for each sample, with each point representing one methylated site. C: The number of sequenced and quality-trimmed reads for each sample. D: Numbers of quality-trimmed reads that mapped back to the chloroplast genome of *Saccharina latissima*.


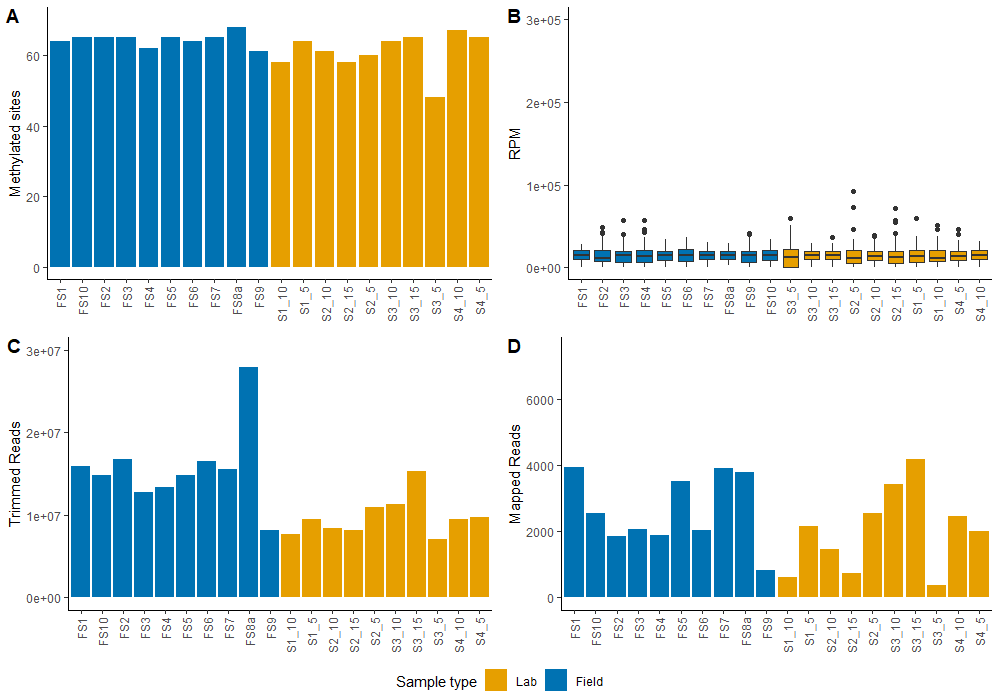


Suppl.Fig. *3*: **Sequencing output and methylation characteristics for each sample from Spitsbergen.** Plots were used for outlier detection for PCA. A: Number of methylated sites (with more than two reads) for each sample. B: Boxplot showing reads per million (RPM) for each sample, with each point representing one methylated site. C: The number of sequenced and quality-trimmed reads for each sample. D: Numbers of quality-trimmed reads that mapped back to the chloroplast genome of *Saccharina latissima*.


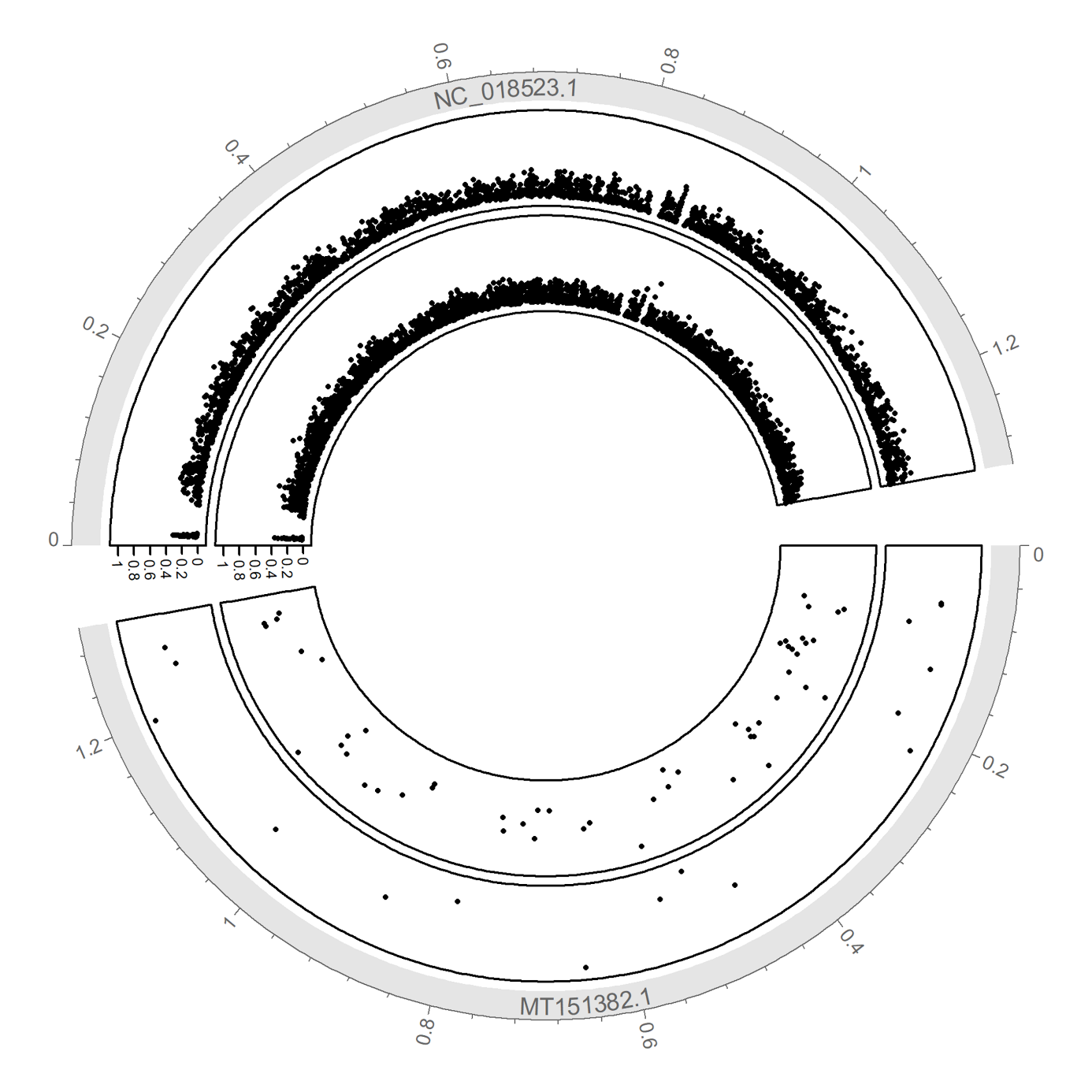


Suppl.Fig. *4:* **Overview of the chloroplast methylome in *S. latissima* (MT151382) and *S. Japonica* (NC_0198523.1).** *S. japonica* data from Teng et al. 2021. Numbers on the outside of the circle represent locations within the genomes. Numbers on y-axes show the methylation levels at each site. The inner circle shows CHG methylation, and the outer circle shows CG methylation. Numbers along the outer circles refer to megabases along the chloroplast genomes.


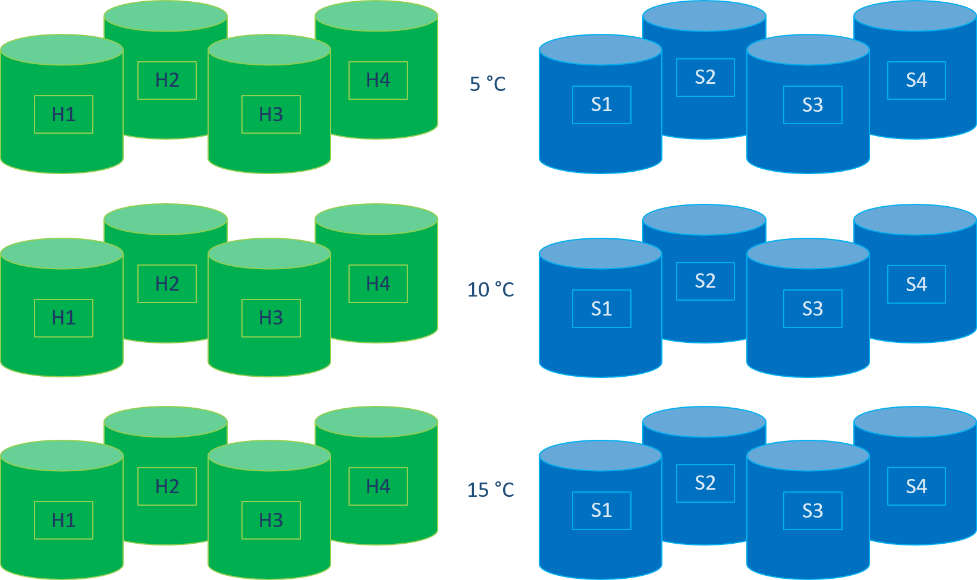
Suppl. Fig. *5*: **Beaker setup at the different temperatures for Helgoland (H1-4) and Spitsbergen (S1-4) samples. For fertilisation scheme, see Suppl. Tab. 1.**


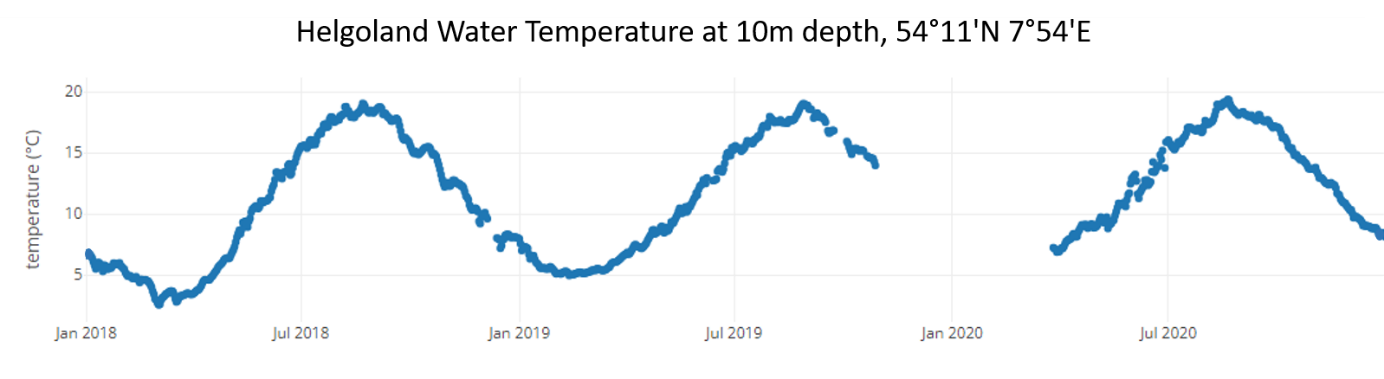


Suppl. Fig. *6*: **Helgoland water temperature at collection depth**, recorded daily via stationary data logger.


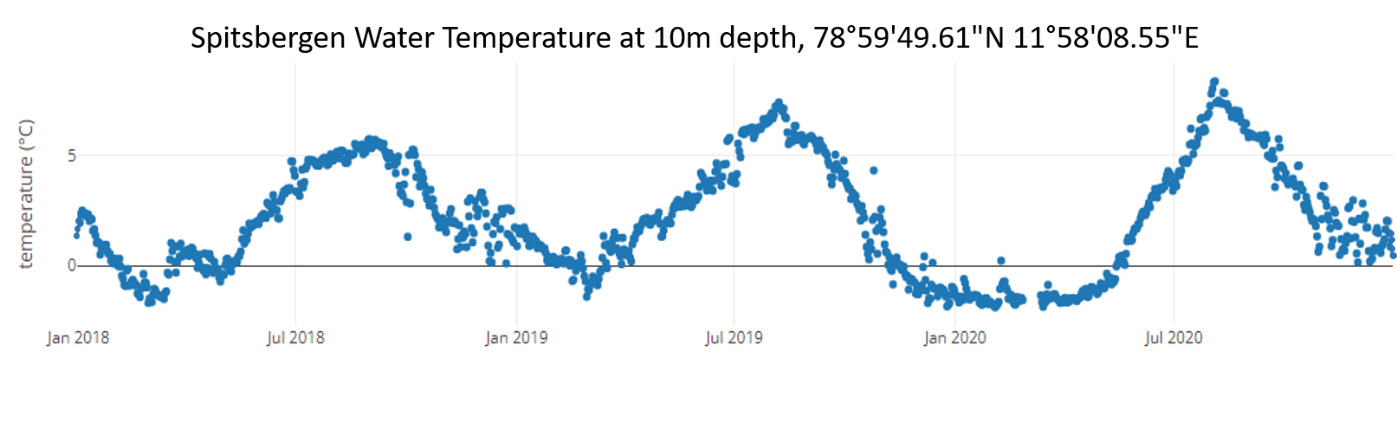
Suppl. Fig. *7*: **Kongsfjorden water temperature at collection depth**, recorded daily via stationary data logger.

### Suppl. Tables

| **Suppl. Table 1: Fertilisation scheme of the laboratory male (♂) and female (♀) gametophyte cultures from Helgoland (HG1-4) and Spitsbergen (SG1-4), resulting in the cultures (Helgoland (H1-4) and Spitsbergen (S1-4) raised under laboratory conditions.** | | | | | | | |
| --- | --- | --- | --- | --- | --- | --- | --- |
| Gametophyte  Cultures | HG2 ♀  X  HG2 ♂ | HG3 ♀  X  HG3 ♂ | HG4 ♀  X  HG4 ♂ | SG1 ♀  X  SG1 ♂ | SG2 ♀  X  SG2 ♂ | SG3 ♀  X  SG3 ♂ | SG34 ♀  X  SG4 ♂ |
| Sporophyte  Culture | H2 | H3 | H4 | S1 | S2 | S3 | S4 |

| Suppl. Table 2  Sites with differential DNA methylation (p < 0.05) between Helgoland and Spitsbergen populations when comparing only field samples. Positive Log2FC values indicate increased methylation in Spitsbergen compared to Helgoland, while negative values indicate higher methylation in Helgoland samples. | | | | | | | | |
| --- | --- | --- | --- | --- | --- | --- | --- | --- |
| Location | Log2FC | Log2FC std error | Adjusted p value | Gene ID | Gene name | Region | Product | Sequence context |
| 21922-21953 | 0.867 | 0.256 | 0.0117 | QKE47428.1 | rpoB | gene | DNA-directed RNA polymerase beta chain | CHG |
| 39358-39389 | 0.776 | 0.222 | 0.0104 | QKE47440.1 | groEL | gene | 60 kDa chaperonin | CHG |
| 75619-75650 | 0.727 | 0.206 | 0.0104 | QKE47479.1 | cbbX | gene | cfxQ-like protein | CHG |
| 55567-55598 | -0.528 | 0.173 | 0.0259 | QKE47459.1 | psaB | gene | photosystem I P700 apoprotein A2 | CHG |
| 51728-51760 | -3.15 | 0.994 | 0.0209 | QKE47458.1 | psaA | promoter | photosystem I P700 apoprotein A1 | CG |
| 55344-55376 | -4.49 | 1.21 | 0.0104 | QKE47459.1 | psaB | gene | photosystem I P700 apoprotein A2 | CG |

| Suppl. Table 3 Sites with differential DNA methylation (p < 0.05) between samples originating from Helgoland or Spitsbergen when comparing only laboratory samples. Positive Log2FC values indicate increased methylation in Spitsbergen compared to Helgoland, while negative values indicate higher methylation in Helgoland samples. | | | | | | | | |
| --- | --- | --- | --- | --- | --- | --- | --- | --- |
| Location | Log2FC | Log2FC std error | Adjusted p value | Gene ID | Gene name | Region | Product | Sequence context |
| 68544-68575 | 1.43 | 0.457 | 0.023 | QKE47473.1 | ycf3 | gene | photosystem I assembly protein | CHG |
| 21922-21953 | 1.18 | 0.285 | 0.000824 | QKE47428.1 | rpoB | gene | DNA-directed RNA polymerase beta chain | CHG |
| 9045-9077 | -5.24 | 0.701 | 2.71E-12 | QKE47418.1 | atpA | gene | ATP synthase CF1 alpha chain | CG |
| 51728-51760 | -5.24 | 1.43 | 0.00438 | QKE47458.1 | psaA | promoter | photosystem I P700 apoprotein A1 | CG |
| 55344-55376 | -5.68 | 0.639 | 4.27E-17 | QKE47459.1 | psaB | gene | photosystem I P700 apoprotein A2 | CG |

| Suppl. Table 4 Sites differentially methylated (p < 0.05) between laboratory and field samples. Positive Log2FC values indicate higher methylation in laboratory samples compared to field samples. | | | | | | | | |
| --- | --- | --- | --- | --- | --- | --- | --- | --- |
| Location | Log2FC | Log2FC std error | Adjusted p value | Gene ID | Gene name | Region | Product | Sequence context |
| 88361-88392 | 2.27 | 0.479 | 0.000137 | QKE47493.1 | rpl3 | gene | 50S ribosomal protein L3 | CHG |
| 15235-15266 | 1.16 | 0.282 | 0.000659 | QKE47426.1 | rpoC2 | gene | DNA-directed RNA polymerase beta' chain | CHG |
| 88373-88404 | 0.987 | 0.298 | 0.00741 | QKE47493.1 | rpl3 | gene | 50S ribosomal protein L3 | CHG |
| 17309-17340 | 0.952 | 0.31 | 0.0124 | QKE47426.1 | rpoC2 | gene | DNA-directed RNA polymerase beta' chain | CHG |
| 64927-64959 | 0.752 | 0.173 | 0.000294 | QKE47469.1 | petA | gene | cytochrome f | CG |
| 34231-34262 | 0.598 | 0.198 | 0.0134 | QKE47436.1 | secA | promoter | preprotein translocase subunit A | CHG |
| 102711-102742 | 0.591 | 0.172 | 0.00654 | QKE47520.1 | tufA | gene | translation elongation factor Tu | CHG |
| 124685-124717 | 0.561 | 0.181 | 0.0123 | QKE47544.1 | ycf39 | gene | NmrA domain-containing protein | CG |
| 46721-46753 | 0.551 | 0.195 | 0.0237 | QKE47451.1 | ycf42 | gene | putative peroxiredoxin ycf42 | CG |
| 32980-33011 | 0.514 | 0.143 | 0.00416 | QKE47436.1 | secA | gene | preprotein translocase subunit A | CHG |
| 102611-102643 | 0.49 | 0.175 | 0.0241 | QKE47520.1 | tufA | gene | translation elongation factor Tu | CG |
| 22542-22574 | 0.439 | 0.161 | 0.0264 | QKE47428.1 | rpoB | gene | DNA-directed RNA polymerase beta chain | CG |
| 75988-76019 | -0.435 | 0.158 | 0.0253 | QKE47479.1 | cbbX | gene | cfxQ-like protein | CHG |
| 104220-104251 | -0.567 | 0.173 | 0.00741 | QKE47523.1 | ftsH | gene | ATP-dependent zinc metalloprotease FtsH | CHG |
| 127333-127364 | -0.641 | 0.191 | 0.00741 | QKE47546.1 | ilvB | gene | acetolactate synthase large subunit | CHG |
| 95574-95605 | -0.728 | 0.16 | 0.000183 | QKE47509.1 | rps5 | gene | 30S ribosomal protein S5 | CHG |

| **Suppl.Table *5:* Statistics results of the epigenetic distance analysis.** | | | | | |
| --- | --- | --- | --- | --- | --- |
| **Epidistance field/lab test results** | | |  | **Euclidean distance** | |
| **Wilcoxon rank sum test** |  | *statistic* | 10499 |  | **Mean dist.** |
|  |  | *p* | 0.0000000173 | *Field* | 153 |
|  |  | *p.signif* | **** | *Lab* | 219 |
| **Shapiro-Wilks test** | *Field* | *statistic (W)* | 0.945 |  |  |
|  |  | *p* | 0.00000106 |  |  |
|  | *Lab* | *statistic (W)* | 0.932 |  |  |
|  |  | *p* | 0.0000000264 |  |  |
|  |  |  |  |  |  |
| **Epidistance Helgoland/Spitsbergen test results** | | |  | **Euclidean distance** | |
| **Wilcoxon rank sum test** |  | *statistic* | 10330 |  | **Mean dist.** |
|  |  | *p* | 0.00000000231 | *Helgoland* | 171 |
|  |  | *p.signif* | **** | *Spitsbergen* | 190 |
| **Shapiro-Wilks test** | *Helgoland* | *statistic (W)* | 0.965 |  |  |
|  |  | *p* | 0.0000305 |  |  |
|  | *Spitsbergen* | *statistic (W)* | 0.957 |  |  |
|  |  | *p* | 0.0000172 |  |  |

| **Suppl.Table *6*** see excel file ‘SupplTab6_7_Fig3_4_Tab2’ sheet ‘ST6 Origins’ |
| --- |
| **Suppl.Table *7*** see excel file ‘ SupplTab6_7_Fig3_4_Tab2’ sheet ‘ST7 Temperature' |
